# Feasibility, Acceptability, and Outcomes of a Yoga-Based Meditation Intervention for Hospice Professionals to Combat Burnout

**DOI:** 10.3390/ijerph18052515

**Published:** 2021-03-03

**Authors:** Carrie Heeter, Marcel Allbritton, Rebecca Lehto, Patrick Miller, Patricia McDaniel, Michael Paletta

**Affiliations:** 1 Department of Media and Information, Michigan State University, East Lansing, MI 48823, USA; 2 Core Resonance Works, New Orleans, LA 70119, USA; marcel.allbritton@me.com; 3 School of Nursing, Michigan State University, East Lansing, MI 48823, USA; lehtor@msu.edu; 4 Northstar Care Community, Ann Arbor, MI 48130, USA; pmiller@hom.org (P.M.); pmcdaniel@hom.org (P.M.); mpaletta@hom.org (M.P.)

**Keywords:** meditation, burnout, interoception

## Abstract

(1) Background. This research examined the feasibility, acceptability and outcomes of delivering a 6-week yoga-based meditation intervention to clinical teams of hospice professionals (HPs) at a large non-profit hospice organization. The intervention was designed to increase mind-body integration and combat burnout. This article was written for different audiences, including research scientists who study interoception, burnout, meditation, or yoga, designers of meditation interventions, and hospice organizations looking for ways to mitigate HP burnout. (2) Methods. The intervention was launched within clinical teams, beginning with a half-hour online introduction to the program and exposure to the week 1 meditation at each team’s monthly all-staff meeting. Throughout the program, HPs could access the meditations on their own via their workplace computers, tablets, and smartphones. Online pre- and post-intervention surveys were submitted by 151 HPs, 76 of whom were exposed to the intervention and completed both surveys. The surveys assessed burnout using the Professional Fulfillment Index and mind-body integration using the Multidimensional Assessment of Interoceptive Awareness scales. (3) Results. Two-thirds of HPs who were present at a staff meeting where the program was introduced went on to do a meditation on their own at least once. Half of HPs expressed a desire to continue with access to the meditations after the 6-week program ended. Due to COVID-19 work from home restrictions, three-fourth of HPs did a meditation at home, 29% in a car between patient visits (not while driving), and 23% at the office. Higher interoceptive awareness was significantly related to lower burnout, particularly lower work exhaustion. Meditation frequency was significantly related to higher interoceptive awareness but not to burnout. Interpersonal disengagement was rare and temporary. (4) Conclusions. Findings showed that the yoga-based meditation intervention was feasible and acceptable and associated with higher interoceptive awareness. The results point to a role for interoceptive awareness in reducing the risk for burnout.

## 1. Introduction

The field research presented in this article examined feasibility, acceptability and outcomes of delivering a yoga-based meditation intervention for hospice professionals (HPs) at a large non-profit hospice organization to combat burnout, a syndrome characterized by mental and physical exhaustion and workplace negativity. Interventions are critically needed to combat the threat of burnout among HPs in order to support the quality of care they are able to provide and to support their personal health and wellbeing. The intervention adapted the tools of yoga to design meditations that promote mind–body integration and reduce the risk of burnout and for hospice workers. We offer a rationale for why yoga-based meditation helps combat burnout and we provide intervention and delivery strategy details, utilization data and outcomes. This thorough explication lays the groundwork for hospice organizations to consider their own intervention strategies and enables burnout and meditation researchers to build upon this study.

In the first four sections, we elaborate on the problem of burnout for HPs. We review prior research on meditation interventions to combat burnout in healthcare professionals and HPs. We explain what we mean by yoga-based meditation. We apply theory and research on mechanisms of effect of yoga to explain why yoga-based meditations might improve mind-body integration and combat burnout.

Then, in the methods section, we clarify important details of the meditation intervention, explain how the intervention delivery strategy was designed to leverage organizational structure and employee work patterns, and discuss study design and instruments used to assess feasibility, accessibility, and outcomes. We report participant demographics.

In the results section, we present feasibility and accessibility findings, including exposure to the meditation intervention and use of the meditations among HPs who were introduced to the program. We report data on meditation use context (home, office and car). Finally, overall pre- and post-intervention data are reported for the burnout and interoceptive awareness scales followed by analyses of the relationship between meditation usage frequency and target outcomes (reducing burnout and increasing mind-body integration).

In the discussion section, we reflect on contributions of the study, including implications for theory, research, and the hospice industry. We also consider study limitations and offer recommendations for future research on yoga-based meditation burnout interventions. The conclusions section summarizes the main achievements of the study.

## 2. HP Burnout and Mind-Body Interventions

Prior research shows that burnout is a serious and pervasive challenge for healthcare professionals overall, including nurses, physicians, and medical school students [1]. Burnout in HPs has also been identified to be a serious problem, compounded by workload stressors and administrative demands [2]. Hospice work is characterized by a multidisciplinary team approach, where interprofessional communication and seamless coordination are essential to ensure optimal delivery of care. There has been careful attention paid to the stressors that working in a hospice and/or palliative care environment places on these essential health care workers over time. Much of this research has been conducted using survey methodology and has depicted the prevalence of burnout in the HP population [3,4,5,6]. Surveys of more than 3500 palliative care and HPs in the US spanning 5 years estimate average burnout rates of 38.7% [7].

Burnout can be addressed through both personal and organizational interventions. Organizational burnout interventions seek to modify workplace values, processes and practices. It is critical to address organizational factors that contribute to burnout. Personal interventions do not replace the need for organization change. These are not mutually exclusive. One of the recommended steps organizations can take is to provide resources to promote HP self-care by offering personal interventions [8].

Mind–body interventions (MBIs) have been evaluated as methods to improve the self-management of stress since the introduction of Mindfulness-Based Stress Reduction (MBSR) by Kabat-Zinn in the 1990s [9]. MBIs can include all types of meditation, relaxation and breathing techniques, yoga, tai chi, qigong, hypnosis, biofeedback, and more [10]. In healthcare, MBIs have been tested as interventions to help professional providers manage work-related stressors [11].

MBIs are often time consuming and a poor fit for busy healthcare professionals. Several healthcare professional studies have been conducted that incorporated the Mindfulness-Based Stress Reduction (MRBR) program [11,12]. MBSR, in its origins, was a group-based 8-week program that incorporated both informal and formal practices. The program included meeting for 2 h per week as a group, an all-day retreat, and daily homework [9]. Attrition rates in the 8-week MBSR program with a sample size of 38 healthcare professionals were reported to be 44%, even with a self-selected sample [13]. Given the demands of the original MBSR program, MBSR-like interventions for health care professionals have attempted to shorten the program, including variations of 1–3 full days [14], and 4 weeks of regular synchronous online sessions with homework [12,15].

MBSR is the most-studied MBI, but it is actually a lengthy amalgam of disparate secularized Buddhist meditations, mindful yoga, and cognitive behavioral practices. Studies of MBSR interventions are unable to separate which of the many techniques are responsible for stress reduction. The mindfulness meditation app, Headspace, developed by a former Buddhist monk, has been studied as a brief intervention to reduce healthcare provider burnout and stress. There are thousands of meditation apps. Headspace consistently ranks among the top five meditation apps [16,17,18] and the company actively supports external research and conducts its own research. Unlike MBSR, using Headspace as an intervention requires minimal time and has no group component or live sessions. Additionally, unlike MBSR, Headspace uses a single consistent meditation modality (mindfulness meditations) rather than an amalgam of techniques.

In research using Headspace with healthcare professionals, participants downloaded the app and did the 10-day, 10 min a day introduction to mindfulness meditation. Some took up to one month to complete the 10 sessions [19,20]. The introductory series purports to teach how to meditate (by sitting in a quiet place, closing the eyes, calming the mind, focusing on the breath, and letting thoughts and feelings come and go [21]).

Novice pediatric nurses who used the Headspace app’s 10 day sequence showed marginally more compassion satisfaction and marginally less burnout than nurses who did traditionally delivered meditation training [19]. Among medical school students, perceived stress decreased and general wellbeing increased among participants in the Headspace meditation app group, compared to a control group, which listened to 10 min autobiographical audio segments about the Headspace founder’s experiences with Buddhism [20].

The MBI intervention for HPs studied in this article is grounded in yoga-based meditation, a form of somatic, movement-based meditation. As will be discussed in subsequent sections, the process, mechanisms and effects of this meditation approach differ from mindfulness meditation. The current research builds upon a study of an earlier, app-based version of the same yoga-based meditation program for HPs [22]. In the initial research, one third of HPs at a medium sized hospice organization who were invited to take part in the program downloaded the app and did a meditation at least two times per week for six weeks. The entire organization participated at the same time. Participants were rewarded USD 20 for each week in which they meditated at least twice. The pre-post study (n = 36) found that burnout was already very low (below 26 on the Professional Quality of Life instrument [23]). Significant though small pre-post survey improvements were found in burnout and compassion fatigue. Significant large pre-post survey improvements were found in the attention regulation, emotional awareness, self-regulation, body noticing, body listening, body trusting dimensions of interoceptive awareness [22]. The study showed that the yoga-based meditation helped HPs cultivate heightened interoceptive awareness and improved mental focus—factors that can change how stressful events are experienced and responded to [22]. Focus group interviews detailed experiential changes that HPs attributed to doing the meditations and affirmed the importance of social support that came from the entire staff going through the program at the same time [24].

The current study tested a revised version of the MBI and an intervention delivery strategy tailored to the needs and affordances of a large hospice organization. HPs were not compensated for doing the meditations. Thus, the uptake of the intervention served as an indicator of its acceptability.

Research on MBI interventions for hospice and palliative care employees tend to show short-term improvements in wellbeing, but many of the studies have low quality of treatment fidelity, small samples, high attrition, or a lack of theoretical consideration of what are the essential “ingredients” that underpin successful interventions [11,25,26]. The meditation components are rarely described in detail and interventions varied in terms of delivery, length, schedule, type of instructor, practice requirements, resources provided, and context. Our study advances the understanding of yoga-based meditation and burnout, informs consideration of MBIs to address HP burnout, and addresses some of the weaknesses of prior MBI research.

## 3. Yoga-Based Meditations as a Burnout Intervention

In this section we explain what we mean by yoga-based meditation and offer a rationale as to why practicing yoga-based meditations designed to promote mind–body integration might help prevent or alleviate the symptoms of HP burnout.

To support the rigorous interpretation of meditation research findings, researchers must precisely define the characteristics of the meditation intervention and the expertise of the intervention developers [27]. The meditation intervention in this study was designed by study authors Allbritton and Heeter, who are experts in meditation and trained researchers with PhDs. Our experience and background in meditation come from the perspective of Viniyoga and Yoga Therapy, originating from Sri Krishnamacharya and TKV Desikachar [28,29,30,31]. Allbritton has studied Viniyoga for over 15 years and is a practicing C-IAYT clinical yoga therapist. Heeter is a professor of user experience and serious game design and an RYS 200 certified yoga and meditation teacher who has been studying and researching meditation for 9 years.

Meditation is a popular form of self-care. A 2017 national study of complimentary care found that 14.2% of U.S. adults reported using meditation [32]. Approaches to meditation that together comprise that 14.2% include mantra meditation, mindfulness meditation, transcendental meditation, guided imagery, progressive relaxation, and spiritual meditation as well as meditation that is part of yoga, tai chi, and qi-gong [33]. Each specific meditation approach is associated with a small fraction of overall meditation practitioners. By comparison, 14.3% of US adults reported doing yoga, including 19.8% of adult women [32].

Different MBI approaches engage the human system in different ways [34]. Yoga-based meditation practices draw upon the tools of yoga (such as attention, breath, physical movement, and meditation objects [35]). The selection and sequencing of steps in a practice are informed by goals for the practice, needs and characteristics of who will be doing the practice, and principles of yoga [36,37,38]. Although the range of possible practices is endless, yoga-based practices typically involve directing attention inward to breath, body, controlled movement, and other internal bodily sensations and feelings [31].

**Figure 1:**
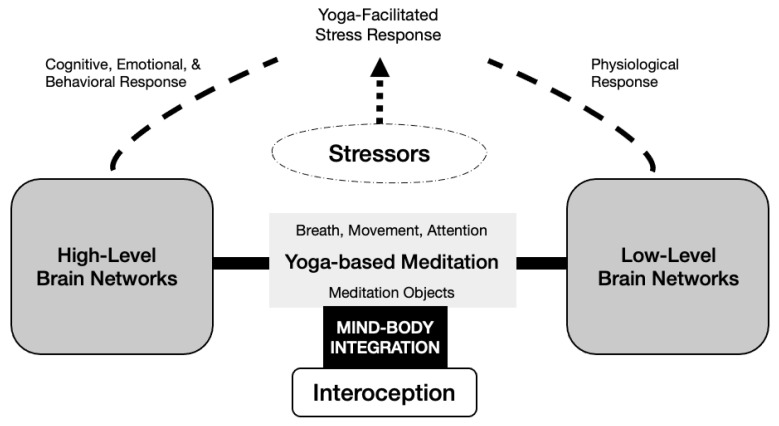
Yoga-based meditation facilitates mind–body integration.

Theories of mechanisms of the effects of yoga suggest that yoga-based meditation supports psychological and physical well-being through combined effects on high level and low-level brain network functioning. (See Figure 1.) In yoga-based meditation, high-level executive function directs attention toward interoceptive signals as they are occurring in the body during breathing, movement and focus on a meditation object while withholding or redirection attention away from mind wandering. Mind–body integration is supported by engaging interoception [14,39,40], the complex iterative process of noticing and appraising and responding to signals originating within the body [34,41]. Slow, controlled movement and breathing and attention to present moment interoceptive bodily sensations and feelings can help quiet the mind, calm the human system and build capacity for interoception [39,42].

Over time, practicing yoga-based meditation supports the inhibition of negative cognitive, emotional, and behavioral responses to stressful events, initially through conscious restraint [14,39,40]. For example, by noticing and self-regulating negative emotions and quieting self-talk. Over time the inhibition becomes automatic. Similarly, autonomic physiological responses to stress during yoga-based meditation gradually transfer beyond the meditation practice period, reducing inflammation, chronic stress response, lowering heart rate, and reducing reactivity [14,39,40]. Yoga-based meditation improves high-level and low-level brain network responses to stressors.

The best way to experientially understand the meditations in the intervention is to spend 12 min actually doing the week 1 calming meditation, which is available as Supplementary Video S1 of this article. Each of the steps in all of the yoga-based meditations that comprise the intervention engage interoceptive awareness and help reduce mind wandering and rumination. The yoga-based meditations in the current study were designed with a long-term goal of helping to inhibit unhealthy responses to stress by training interoception and promoting mind–body integration. There are other general and specific goals of each meditation but activating and training interoception is a prominent and omnipresent function. The meditations are described in detail in Section 5.1.

## 4. Intervention Outcomes

In this section, we introduce two categories of outcomes of the meditation program (burnout and mind–body integration) and discuss why doing yoga-based meditations might bring about desirable changes for HPs.

### 4.1. Burnout and Yoga-Based Meditation

To assess burnout, the study used the brief Stanford Professional Fulfilment Index (PFI) to capture elements of HP distress and dimensions of wellbeing over the last two weeks. The 16-item PFI measure is suited to assessing changes over time in work exhaustion, interpersonal disengagement and professional fulfilment [43]. The index has high reliability and convergent validity.

Work exhaustion combines feeling physically and emotionally exhausted at work, lacking enthusiasm for work, and feeling a sense of dread about the work one has to do. Interpersonal disengagement combines feeling less empathetic to one’s patients and to one’s colleagues, being less interested in talking with patients, less connected with patients and less sensitive to others’ feelings/emotions. Work exhaustion and interpersonal disengagement can be merged to measure overall burnout. The third dimension of the PFI, professional fulfilment, measures feeling satisfaction, feeling worthwhile, meaningfulness and having a sense that one is making a professional contribution.

### 4.2. Mind–Body Integration, Interoception, and Yoga-Based Meditation

As discussed in Section 3, theories of the mechanisms underpinning the beneficial effects of yoga-based meditation predict (1) beneficial impacts of regular practice on high level and low-level brain functions and (2) better integration of mind and body through interoception. Interoceptive awareness is one aspect of interoception, which theories of yoga point to as central to mind–body integration and to changes in how the human system responds to stress. The Multidimensional Assessment of Interoceptive Awareness (MAIA) is a survey instrument designed to measure the effects of meditation on multiple dimensions of interoceptive awareness [12,44,45]. The scale was developed by a diverse team of scientists and practitioners with expertise in different meditation traditions, including yoga, mindfulness, qigong, somatic massage, and mindfulness.

The yoga-based meditations in this study intervention were designed to activate and train interoceptive awareness most closely related to six of the eight MAIA subscales. (The other two subscales, non-distracting and not-worrying, ask about reacting to sensations of discomfort or pain, which our intervention does not address.) Below, we present MAIA’s explanation of the six subscales and offer two example questions from each subscale [45]:

- Self-regulation (ability to regulate distress by attention to bodily sensations). Example questions: When I bring awareness to my body, I feel a sense of calm; When I am caught up in thoughts, I can calm my mind by focusing on my body/breathing.
- Emotional awareness (awareness of the connection between body sensations and emotional state). Example statements: I notice how my body changes when I am angry; I notice that my body feels different after a peaceful experience.
- Attention regulation (ability to sustain and control attention to body sensations). Example statements: I can maintain awareness of my inner bodily sensations even when there is a lot going on around me; I can return awareness to my body if I am distracted.
- Body noticing (awareness of uncomfortable, comfortable, and neutral bodily sensations). Example statements: When I am tense I notice where the tension is located in my body; I notice changes in my breathing, such as whether it slows down or speeds up.
- Body listening (active listening to the body for insight). Example statements: I listen for information from my body about my emotional state; When I am upset, I take time to explore how my body feels.
- Body trusting (experience of trusting one’s body as safe and trustworthy). Example statements: I feel my body is a safe place; I trust my bodily sensations.

The categories, summaries, and example questions illustrate what each subscale measures, and suggest ways that interoceptive awareness contributes to wellbeing, stress management, and combatting burnout.

## 5. Methods

The methods section is organized in detail about the yoga-based meditation intervention, the intervention delivery strategy, and survey instruments. Next, sample size and demographics are presented.

### 5.1. Details about the Intervention

To support future meta-analyses and rigorous research, meditation researchers [37] and yoga researchers [46,47] advocate for a thorough description of the process and intended outcomes for meditation interventions used in research. We begin by describing the overall structure of the intervention and then go into detail about the content for each week.

**Figure 2:**
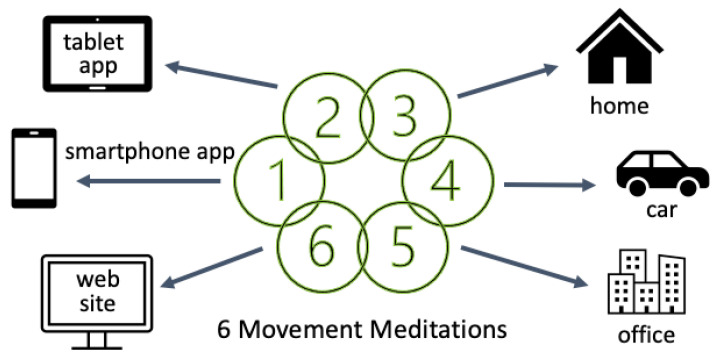
Ways HPs Could Access the Meditations.

The intervention offered one new 12-min yoga-based meditation recording per week for six weeks, half guided by a male meditation teacher (Allbritton) and half by a female meditation teacher (Heeter). HPs could access the meditations by logging in to the program website or by opening an app on the tablets and smartphones they used for hospice work. The program website had a page for each week with the meditation video (audio guidance and video of stick figures illustrating each step), a “how and why this works” video explaining principles of yoga-based meditation that have implications for daily life (see Figure 2), and additional messaging to motivate regular meditation practice. The internal app only included meditation audios.

Yoga-based meditations consist of a series of steps that draw upon the tools of yoga (such as movement, breath, and meditation objects). Table 1, Table 2 and Table 3 lists the specific movements, breathing, and attentional focus used in each meditation to enable readers expert in yoga to understand the intervention. These steps were selected and sequenced to help bring about the intended changes in the human system. For the meditations in the intervention, there were many design constraints. The duration had to be under 12 min. The practices, including clear instructions and amount of time in any step, had to be accessible to beginners who have never done yoga before. The practices were done seated comfortably in a chair. Movements needed to be gentle and participants were reminded to move only as far as is comfortable, to avoid potential stress or strain.

Each meditation had unique goals. For example, the steps and sequencing of steps in the calming meditation were chosen with the goal of relaxing the body and quieting the mind. Aligning gentle movements with breath gave the mind something to do other than think about itself. Early steps involved larger movements (such as bringing the arms up from the front toward the ceiling). As the meditation progressed, the mind and body became calmer, allowing movements to be more subtle. As the meditation progressed, participants were guided to move a little slower on exhale. Additionally, in the later steps, to pause for a moment or two at the end of each exhale. These exhale techniques support calming. The general intended direction of effect for each meditation is implied by its name: calming, peaceful feeling, nature, stability, releasing, and cleansing waves.

All of the meditations in the intervention shared objectives of engaging interoception and promoting mind–body integration. Theoretical models and reviews of research explain how and why yoga-based meditations promote mind–body integration [14,34,35,39]. For example, synchronizing slow, controlled gentle movements with inhale and exhale requires coordination, tracking the body’s location in space, and attention to both breath and movement. Executive function is involved in learning each movement and breath sequence. Interoceptive attention is engaged with kinesthetic, proprioceptive and spatial sensations [39].

Table 1 lists all of the gentle movements were part of the intervention, noting which of the meditations that movement appeared in. The first two movements and postures were part of all six meditations. Others were unique to particular meditations. For example, arm extension out from the chest, palms facing outward, supports releasing. Leaning forward and back and finding a position that feels most stable supports stability. The movements in yoga-based meditations were chosen to support the function of that meditation.

Attention to and regulation of breath are also tools of yoga-based meditations. Table 2 shows that, as previously discussed, every meditation includes aligning movement with breath. Different yoga breathing techniques (extending exhale, pausing after exhale, visualization linked to inhale and exhale), were used to support the focus of particular meditations. The first two breath techniques (free observed breath and aligning movement with breath) were part of all of the meditations.

Meditation objects are another tool used in yoga-based meditation. A meditation object is an object the mind focuses on. Table 3 shows meditation objects for the intervention meditations. Meditation objects can help the meditator strengthen their connection to a the object or quality (such as calm, peaceful, stable [48]). In the nature meditation, participants called to mind the experience of being in a favorite place in nature, activating interoceptive feelings of spending time in nature and of being in a good-feeling place. The releasing meditation brought forward the idea of letting go of things the HP was holding on to that were no longer needed (perhaps tension or tightness, perhaps events from a stressful day). Additionally, the cleansing waves meditation brought forward the idea of cleansing and the calming rhythm of breath as gentle waves.

The program web site page content and videos for each week emphasized a learning principle that showed how what HPs were doing in the meditation can translate into daily life. Table 4 lists the principle for each week.

These principles apply to all six meditations, but are introduced one week at a time, in logical order that is consistent with the particular meditation for that week and with the users’ growing experience with this meditation modality. Doing the week 1 calming meditation reinforced the principle that “yoga-based meditations are tools you can use to help change how you feel,” because this aligned with their experience of doing the meditation. At the beginning and end of each meditation, HPs were guided to notice how they feel. To check in with themselves. The principle for week 2 was that “checking in with yourself” is a skill, in meditation and in life. Regular practice of these yoga-based meditations built a capacity to check in with oneself. The “how and why this works” video for week 2 is included as Supplementary Video S2 with this article.

### 5.2. Meditation Program Delivery

The impact of a burnout intervention for HPs depends upon both the design of the meditation program itself and also on the implementation approach and resulting uptake of the meditations by HPs. In this section, we explain the program delivery decisions and the organizational strategies intended to motivate and facilitate social support for doing the meditations.

#### 5.2.1. Adapting Program Delivery to the Needs of Clinical HPs

The practice of clinical hospice work has implications for how to deliver a yoga-based meditation intervention. Clinical HPs are a mobile workforce who deliver onsite service to dying patients and their families and offer virtual care support by phone. The mix of in person and phone support varies by role, with hospice aides and nurses providing the most in-home care and chaplains and social workers engaging in more phone support. All patient care involves continuous need to access and update Electronic Medical Records (EMR). The hospice organization provides clinicians with secure tablets and smartphones to use for EMR and internal communication systems.

The original plan was to offer the yoga-based meditation program by providing HPs with login accounts to the mobile friendly program web site. Depending on the location of patient homes, internet access can be unreliable, especially in remote, low population density regions. To make access to the meditations as easy as possible and to eliminate the need for internet access, the hospice IT department created an internal app that included audio of all six meditations, which IT “pushed out” to all workplace tablets and smartphones. Thus, we took advantage of omnipresent secure tablets and smartphones that were deeply integrated into HP workflow, making it easy to access the meditations. MP3 audios of the meditations were used in the app instead of the videos because video files would have taken more storage space on workplace mobile devices. Since the meditations were done with eyes closed, the only difference was that HPs could not open their eyes to see a diagram of a movement if they were uncertain what to do.

Thus, the yoga-based meditation intervention was accessible via the program web site. HPs could also do the meditations by opening the app on their tablet or smartphone and playing the meditation audio, with no need for a log-in or internet access. There were three venues where HPs could do the meditations: at the office, at home, and in their cars (not while driving!) before or after patient visits (see Figure 2).

#### 5.2.2. Leveraging Organizational Structure to Foster Community and Encourage Adoption and Use

Here we discuss program delivery decisions informed by the structure of the large hospice organization to help HPs value the program, motivate them to do the meditations, and harness peer and management involvement for social support.

The 6-week intervention was offered in the context of clinical teams, so the intervention would be a shared experience among co-workers. The non-profit hospice we studied served dying patients in 62 counties across the state. The hospice was organized into geographic service regions, and within those regions into local clinical teams that deliver hospice care support directly to patients and their families. Each team was led by an operations manager who coordinated the work of nurses, social workers, hospice aides, and chaplains. (There were other services and teams across the hospice organization, but the focus of our research was on the clinical teams).

The implementation approach elicited leadership buy-in to demonstrate the institutional valuing of the importance of the intervention. Yoga-based meditation program coordinators introduced the program at an online meeting to the hospice organization’s Executive Leadership team, and the executives did the week 1 meditation (calming) together, then discussed plans for the program. Then, the program was introduced at an online meeting to the clinical directors, and they did the week 1 meditation together. Next, online introduction sessions were held with each clinical director and the clinical operations managers of the teams she supervised, and that group did the week 1 calming meditation together. Finally, for each clinical operation manager’s team at their regularly scheduled monthly team all-staff meeting, a yoga-based meditation program coordinator joined the meeting online, introduced the program to the team, and helped them locate the app on their tablets or smartphones.

The six-week program was deployed separately to each team tied to their monthly all-staff meeting schedules. Every participant who was able to attend their team’s monthly online all-staff meeting was introduced to the 12-min week 1 meditation during the meeting. The operations manager forwarded an email to their team each week throughout the program, introducing the new meditation for the week, pointing out the principle for the week, and encouraging use.

At the introductory online session after the team did the calming meditation, the researcher encouraged HPs to do the meditation on their own and showed a slide suggesting ways HPs could use the meditations:

- Do meditations when teams are together throughout the 6 weeks.
- Try to do the meditation on at least one (ideally more) other days each week on your own.
- In the car (while parked, not while driving!).
- When you first get up.
- Before you go to bed.
- After or before seeing a client.

Thus, HPs could do the 12 min meditations during working hours or on their own time. The research protocol did not reward HPs for meditation frequency because we wanted to measure acceptability and adoption of the intervention on its own merit. Teams were rewarded with a team gift card based on the percent of team members who submitted surveys.

### 5.3. Measures

Here we explain how feasibility, acceptability and intervention outcomes were measured.

#### 5.3.1. Meditation Use, Feasibility, and Acceptability

A primary aim of this research was to assesses the feasibility and acceptability of the yoga-based meditation intervention. In this section, we define and operationalize feasibility and acceptability.

A consequence of making the meditations available on a local app on workplace smartphones and tablets was that the researchers had to rely on self-report instead of automatically tracking meditation usage. The program web site did track all meditation use for each logged in user, but almost all HP meditation use ended up being via the local app. Usage tracking of the custom app was beyond the scope of time and effort available to the project.

Feasibility refers to the practicality and viability of the of the team-based intervention delivery strategy as a means to reach clinical HPs with the meditation program [49]. Feasibility was assessed using a self-report on the post-survey. The post-survey asked whether the HP had done any of the meditations. Since the program was introduced to the team, all-staff meetings in which the first week meditation was played, doing at least one meditation showed that the HP had been reached by the intervention.

Acceptability refers to how recipients react to the intervention [49]. Acceptability was assessed using self-report on the post-survey. Respondents were asked how many times they did a meditation, what platforms they used (tablet, phone, computer), where they did meditations (home, car, office), and whether they would be interested in continuing to have access to the meditations now that the 6-week program was over.

#### 5.3.2. Outcomes (Burnout and Mind–Body Integration)

A secondary research aim of this study was to examine whether the intervention resulted in changes in burnout or mind–body integration among participating HPs. In this section, we introduce the instruments used to measure these outcomes. Details about these survey items were discussed earlier in Section 4, Intervention Outcomes.

Burnout was assessed on the pre- and post-surveys using the Professional Fulfillment Index which measures professional fulfilment, work exhaustion, and interpersonal disengagement [43]. This index was described in Section 4.2.

Mind–body integration was assessed on the pre- and post-surveys using six dimensions of interoceptive awareness from the MAIA scale: Self-regulation, attention regulation, emotional awareness, body noticing, body listening, and body trusting [44,45]. These scales were described in Section 4.2.

### 5.4. Study Protocol, Sample Characteristics, and Overall Burnout and Interoceptive Awareness Levels

#### 5.4.1. Protocol

Here we describe in detail the study protocol including how participation was organized within regional teams, team-based rewards for participation, IRB approvals, consent procedures and the study schematic.

Clinical care delivery at the hospice we studied is managed by 20 different regional teams, each with its own operations manager, nurses, hospice aides, social workers, and chaplains/spiritual care professionals. The intervention was deployed to all clinical care teams, one team at a time.

Teams were rewarded with a gift card where the amount was determined by the proportion of team members who submitted a pre-survey (up to USD 100) and the proportion of team members who submitted a post-survey (also up to USD 100). There were no individual or team incentives for doing the meditations.

The online pre-post survey research design and intervention delivery protocols used in the research were reviewed by the Michigan State University IRB and received an exempt determination (category 3i(b)), STUDY00003891. The pre- and post-surveys began with an online consent form. Consenting HPs then moved to the survey questions.

As we stated in the Conflict of Interest section for this article, the meditation intervention is a commercial product and the first two authors are associated with the company. The consent form included the following statement: We want to disclose that Yoga Mind Tools is a commercial program, and the creators (Carrie and Marcel) may personally benefit from future sales if this trial is successful.

The meditation intervention was delivered to 11 clinical care delivery teams in spring (Wave 1) and 7 teams in fall (Wave 2). Two other teams scheduled for fall participation had to postpone starting the program and were not included in the research.

A mandatory statewide COVID-19 shut down began near the end of Wave 1, upending data collection. Before COVID-19, clinical HPs worked either on the road or at the office. COVID-19 restrictions forced an immediate shift to work from home instead of working from the office. In-home patient visits still happened, but with new safety precautions. Some services previously offered in person occurred virtually.

Our university IRB shut down all research, and the hospice organization was occupied with the sudden, immediate need to pivot to employees working from home instead of the office and establishing home visit procedures to protect HPs, patients, and their families from exposure to COVID-19. Therefore, post-surveys from the first 11 teams could not be collected. We did continue sending teams weekly emails introducing the new meditation for the week, and some operation managers contacted the PI to report that the meditations were helpful in coping with challenging times. The researchers continued to send the HP teams weekly emails introducing the new meditation for the week.

To give the hospice organization time to normalize to COVID-19 changes, we waited 6 months to start the second wave of deployment to hospice teams. Due to ongoing COVID-19 safety measures throughout the Wave 2 research period, HPs logged in to the all-staff meetings from home instead of meeting in person together as they had before COVID-19. Three teams use Microsoft Teams, and 4 teams use Zoom for their all-staff meetings.

**Figure 3:**
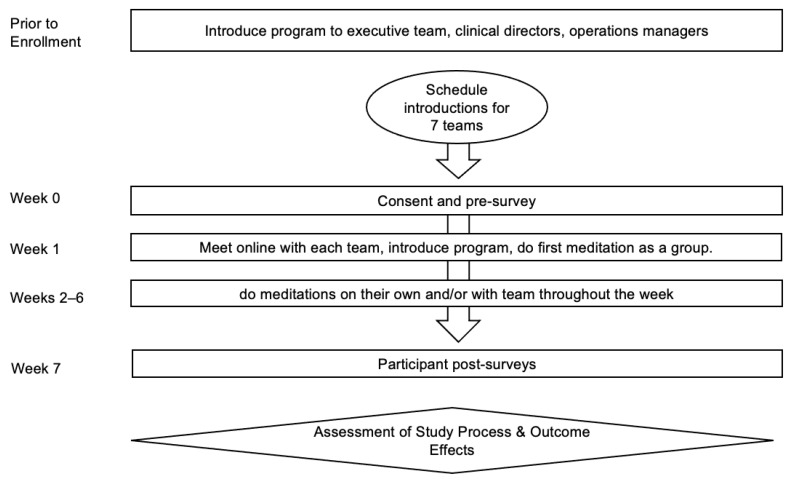
Study Schematic.

Figure 3 shows the Wave 2 study schematic.

#### 5.4.2. Sample Characteristics

Here sample size on the pre- and post-survey response rates and sample characteristics are reported. Survey results describe team characteristics and professional roles of HP study participants. Demographics are estimated based on an earlier survey conducted within the hospice organization.

Pre-surveys were submitted by 127 clinical HP team members and post-surveys were submitted by 116 HPs. Ninety-two pre-post surveys were able to be matched using participant email addresses. This means that 61% (n = 92) of the 151 unique participants submitted both a pre-survey and a post-survey.

The teams participating in the study served patients and families in home hospices. Several teams covered large, sparsely populated counties. Others served medium sized cities and surrounding areas. The size of the seven teams ranged from 7 to 30 members.

HP role was only asked on the presurvey. By role, 43% of pre-survey respondents were nurses and 22% were hospice aides (see Table 5). The remaining roles (social worker, chaplain/spiritual care, manager, administration, and other) each comprised fewer than 10% of respondents.

Demographics were not asked on the surveys, due to a need to keep the survey under 12 min while including the PFI and MAIA items. As a measure of comparison with other hospices, we offer organization-wide demographics from comprehensive survey of all clinical employees at the hospice organization that was conducted in 18 months earlier. That survey showed that 90% of the hospice organization’s clinical employees were female, 90% white, and 90% age 30 or older.

## 6. Results

Results are organized into feasibility and acceptability findings, overall levels of pre- and post-survey burnout and interoceptive awareness, and analysis of the outcomes of meditation use.

### 6.1. Feasibility and Acceptability

Feasibility was measured as the percent of HPs who were able to attend their team’s all-staff meeting where the program was introduced, and the week 1 meditation was played. Acceptability was assessed by examining details of how often, on what platform, and where the movement meditations were done by HPs who were exposed to the intervention. Desire to continue to have access to the meditations after the 6-week intervention ended was another indicator of acceptability.

#### 6.1.1. Feasibility

Among the 116 post-survey respondents, 22% never experienced meditation. (They must have missed the all-staff meeting where the program was introduced, and the first meditation was played.) Thus, the intervention delivery strategy of introducing the program at each team’s monthly all-staff meeting successfully reached 78% (n = 91) of HPs on those teams.

While running the program introduction sessions, it became clear to researchers that locating the app with the meditation audio on tablets and smartphones was not obvious or intuitive. It took time and many HPs attending the introductory online session relied on colleagues who had found the app to show them how to get to it. As will be seen in the next section on acceptability, the majority of meditation usage occurred via smartphone or tablet, not from logging on to the meditation program web site. A conclusion is that it was necessary for HPs to be at the program launch meeting so that they would know how to find the meditations and be able to participate in the intervention.

#### 6.1.2. Acceptability

An important measure of acceptability is whether the yoga-based movement meditations appealed enough to participating HPs that they made time to do the meditations on their own.

Table 6 shows the frequency of meditation use. HPs were asked on the post-survey how many times they did a meditation. Response categories were 0; 1; 2; 3 to 5; 6 to 10; more than 10. Since only one HP did meditations more than 10 times, that response was collapsed into a 6 to 10+ times category. Column 3 is the percentage of all post-survey respondents across all five categories of meditation use. Column 4 includes only HPs who were exposed to the intervention at their team’s all-staff meetings and, therefore, did at least the week 1 meditation at least one time.

Results show that about one third (34%) experienced one meditation, presumably as a group at the online all staff meeting, and never made time to do a meditation again. Conversely, two-thirds of HPs who were exposed to the week 1 meditation did go on to do at least one meditation on their own.

About one-fourth (24%) of HPs who were exposed to the intervention made the effort to do a meditation one time on their own in addition to the all-staff meeting, for a total of two meditation sessions. Nearly one-third (32%) experienced program meditations 3 to 5 times. Nine percent did meditations between 6 and 10 times.

The intervention was acceptable enough that two thirds of HPs who were exposed to the week 1 meditation went on to meditate again on their own. However, only 9% meditated six or more times. Analysis of outcomes in later sections of this report will examine the question of whether enough meditation experience occurred to result in a measurable change in burnout or interoceptive awareness.

Findings about where and how HPs did the meditations confirm the importance of making the intervention directly and easily available on hospice tablets and smartphones in addition to the program website. Three-fourths of post-survey respondents who played a meditation at least one time (n = 91) did so at home. About one-fourth (23%) played at least one meditation at the office. Additionally, 29% did a yoga-based meditation in their car (not while driving). These add to more than 100% because some participants did meditations in more than one location.

Among post-survey respondents who played a meditation more than at the initial all staff meeting (n = 38), 57.9% indicated that they did so using their workplace tablet, and 38.9% used their company smartphone.

There were six different meditations, with a new meditation introduced each week. Participants could do any meditation as often as they wanted. Sixty-two percent of HPs only experienced one of the six meditations; 20% experienced two different meditations; 10% experienced three different meditations; 8% experienced four to six of the six meditations.

A promising indicator of acceptability of the intervention is that, among HPs who did a meditation at least once (n = 91), 48% indicated they would like to continue to have access to the meditations after the 6-week program ended. (HPs who did not do any meditations were not asked whether they would like continued access.)

Meditation use and interest in having continued access varied across the seven teams. The percent of post-survey respondents on each of the seven teams who were exposed to at least one meditation ranges from 60% to 87%, contributing to an average exposure of 79% across all teams. Desire to continue to have access to the meditations after the 6-week program ended was significantly different by team (chi square = 13,449, df = 6, *p* = 0.036) with the percent of team members who wanted continued to have access ranging from a low of 0% to a high of 86% across different teams. This wide variation suggests something related to the team influenced meditation use.

### 6.2. Overall Pre-Post Burnout and Interoceptive Awareness

Here, overall pre-post survey levels of burnout and interoceptive awareness are reported. As described in Section 5.4.2, there were 127 pre-survey responses and 116 post-survey responses. Only 61% (91) of HPs completed both a pre-survey and a post-survey and, as shown in the feasibility and acceptability section, 84% (76) of HPs who completed both surveys were exposed to the intervention.

The next two results sections focus on only those 76 participants who completed both surveys and were exposed to the intervention at least once. Pre-post comparisons provide a sense of levels of HP burnout and interoceptive awareness in week 0 and week 7.

#### 6.2.1. Overall Pre- and Post-Survey Burnout

In this section, baseline pre-survey and post-survey PFI scores on professional fulfilment, work exhaustion, and interpersonal disengagement are reported. The data provide a general sense of the level of burnout among participating HPs in the 2 weeks prior to launching the meditation intervention and one week after the intervention ended. Although all respondents in the results were exposed to the intervention, the tables here do not differentiate HPs who went on to do meditations on their own from those who only experienced the initial meditation at the introductory all-staff session.

Burnout was measured in order to examine effects of participating in the meditation program. Here we report overall pre- and post-survey burnout across all respondents, regardless of meditation use. Table 7 reports overall professional fulfilment, work exhaustion, and interpersonal disengagement data across all respondents. The table includes average score, standard deviation, the percent of employees whose scores qualified as “very high”, and number of respondents.

Responses on the scale can be between a low of 0 and a high of 4. Based on research, PFI developers have determined cut points for what is considered “high” on each of the three subscales [50]. For professional fulfilment, the cut point for classifying respondents as experiencing professional fulfilment is >3.0. For work exhaustion, interpersonal disengagement, and their combination, the cut point classifying respondents as experiencing these forms of burnout is 1.33 [50]. The pre-survey results show that 39% of Wave 2 HPs reported high professional fulfillment, 45% reported high work exhaustion and 14% reported experiencing high interpersonal disengagement.

Pre- and post-survey PFI scores were compared using paired t-tests. Pre-survey professional fulfillment was significantly better (higher) than post-survey professional fulfillment (*t* (76) = 2.713, *p* = 0.008). Pre-survey interpersonal disengagement was significantly worse (higher) than post-survey interpersonal disengagement (*t* (71) = 2.003, *p* = 0.049). Workplace exhaustion and overall burnout were not different between pre- and post-intervention surveys.

The measurement of professional fulfillment and burnout 7 weeks apart (before and after the intervention period) provided a detailed view of how stable or changeable those dimensions of burnout were for this HP study population. Table 8 shows both percent and raw number of HPs classified as high on each PFI scale. The rows represent pre-survey states and the columns represent post survey classifications.

Professional fulfillment was the same in week 0 and week 7 for 72% of HPs. Fifty-nine percent of HPs on the pre-survey did not report high professional fulfilment, while 41% did report high professional fulfillment. On the post-survey, 71% did not and 29% did report high professional fulfillment. Professional fulfilment flipped for 21 individual HPs, with 15 shifting out of high professional fulfillment and 6 shifting into high professional fulfillment. Sixteen HPs reported high professional fulfillment in both week 0 and week 7. Forty-three percent of HPs who reported high professional fulfillment in either survey reported high professional fulfillment in both surveys.

Work exhaustion was the same in week 0 and week 7 for 76% of HPs. Sixty-one percent of HPs on the pre-survey did not report high work exhaustion, while 39% did report high work exhaustion. On the post-survey, 56% did not and 44% did report high work exhaustion. Work exhaustion flipped for 17 individual HPs, with 6 shifting out of high work exhaustion and 11 shifting into high work exhaustion. Twenty-one HPs reported high work exhaustion in both week 0 and week 7. Fifty-five percent of HPs who reported high work exhaustion in either survey reported high work exhaustion in both surveys.

Interpersonal disengagement was the same in week 0 and week 7 for 73% of HPs. Seventy-eight percent of HPs on the pre-survey did not report high interpersonal disengagement, while 22% did report high interpersonal disengagement. On the post-survey, 88% did not and 13% did report high professional fulfillment. Interpersonal disengagement flipped for 19 individual HPs, with 13 shifting out of high interpersonal disengagement and 6 shifting into high interpersonal disengagement. Only three HPs reported high interpersonal disengagement in both week 0 and week 7. Only 13% percent of HPs who reported high interpersonal disengagement in either survey reported high interpersonal disengagement in both surveys.

Of the three PFI scales, work exhaustion was the most persistent across the two time periods, whereas self-reported levels of interpersonal disengagement changed the most.

#### 6.2.2. Overall Pre- and Post-Survey Mind–Body Integration

Here we report overall pre- and post-survey interoceptive awareness across all respondents, regardless of intervention exposure or meditation use. Similar to the burnout data in the previous section, mind–body integration data provide a general sense of the level of the six dimensions of interoceptive awareness among participating HPs prior to launching the meditation intervention and after the intervention ended.

Table 9 reports overall self-regulation, attention regulation, emotional awareness, body noticing, body listening, and body trusting levels and composite overall MAIA scores across all respondents. Overall, MAIA is a composite variable computed by averaging the sum of the scores for each of the six subscales. Table 9 includes average score, standard deviation, and number of respondents. The scores for each MAIA subscale can range from a low of 1 to a high of 6. Paired *t*-tests of pre- and post-survey MAIA scores showed no significant differences.

### 6.3. Outcomes

Here, we examine whether the intervention reduced burnout—specifically, whether meditation frequency was associated with lower burnout. We also look at interrelationships among the four professional fulfillment PFI indices and the six MAIA dimensions of interoceptive awareness.

#### 6.3.1. Meditation, Interoceptive Awareness, and Burnout

Independent sample t-test mean comparisons were used to test whether HPs classified as high on the PFI scales differed in meditation frequency or interoceptive awareness. No relationship was found between meditation frequency and professional fulfillment, workplace exhaustion, or interpersonal disengagement.

Table 10 reports significant differences based on independent sample t-test mean comparisons for post-intervention professional fulfillment. The PFI indices were interdependent. Considering the relationships among the three PFI scales, HPs who reported high post-intervention professional fulfillment were less likely to report experiencing pre-intervention work exhaustion (only 14% of professionally fulfilled HPs had been reporting high work exhaustion, compared to 48% who had not reported professional fulfillment) (*t* = 2.928 (df = 74), *p* = 0.005). None of the HPs who reported high post-intervention professional fulfillment had reported pre-intervention interpersonal disengagement, whereas 30% of HPs who were not experiencing professional fulfillment had reported high interpersonal disengagement. (*t*-tests could not be conducted because one of the two averages was 0).

One of the six MAIA subscales, body trusting, was significantly higher among HPs who were high in professional fulfillment (x = 5.28, s.d. = 0.70) than among those reporting low professional fulfillment (x = 4.30, s.d. = 1.14, *t* = 3.579 (df = 70), *p* = 0.005). (Body trusting refers to experiencing one’s body as safe and trustworthy).

Table 11 reports significant independent sample *t*-test differences in post-intervention professional fulfillment and interoceptive awareness indices comparing HPs experiencing high and low overall burnout. HPs who reported high burnout were significantly less likely to report post-intervention professional fulfillment. Only 6% of HPs with high burnout were high in professionally fulfillment, whereas 39% of HPs who were not experiencing burnout had high professional fulfillment.

Three of the six MAIA subscales (self-regulation, body trusting, and body listening) were significantly lower among HPs who reported high burnout. These findings support the theoretical premise that interoceptive awareness plays a role in mitigating burnout. HPs who had better self-regulation, body trusting, and body listening reported less burnout. They were able to actively listen to their body for insight, to direct attention to bodily sensations, to notice how they were feeling, and to use that internal interoceptive focus as a way to regulate distress.

Table 12 reports significant independent sample t-test differences in post-intervention work exhaustion and interoceptive awareness between HPs experiencing high and low work exhaustion (a subcomponent of overall burnout). HPs who reported high work exhaustion were less likely to report experiencing professional fulfillment (only 24% of work exhausted HPs reported high professional fulfillment, compared to 55% who were not work exhausted). Interpersonal disengagement occurred exclusively among HPs who also reported high work exhausted HPs. Forty-four percent of HPs who were work exhausted also reported high interpersonal disengagement, but only one of the HPs who was not work exhausted reported interpersonal disengagement.

Three of the six MAIA subscales (self-regulation, attention regulation, and body listening) were significantly lower among HPs who reported high work exhaustion.

Table 13 shows significant independent sample *t*-test differences in PFI and interoceptive awareness between HPs experiencing high and low interpersonal disengagement (a subcomponent of overall burnout). Consistent with earlier reporting, high interpersonal disengagement was associated with less professional fulfillment. (*t*-tests could not be conducted because one of the two averages was 0). Work exhaustion was significantly more likely to be present among HPs reporting high interpersonal disengagement (78%) than among HPs not reporting interpersonal disengagement (37%).

None of the six MAIA subscales was significantly different based on level of interpersonal disengagement.

#### 6.3.2. Meditation and Interoceptive Awareness

To test the impact of the intervention on interoceptive awareness, one-way ANOVAs were run to compare frequency of doing the meditations with MAIA scores. For these analyses, all HPs who completed a post-survey and were exposed to the intervention were included (n = 79). Three groups of HPs were compared: those who were exposed to a meditation at all-staff meeting but never did a meditation on their own (34%), HPs who did a meditation on their own one time in addition to the all-staff meeting (22%), and those who did a meditation two or more times on their own (44%).

Table 14 shows the number of participants and average meditation frequency of these three groups. By definition the average times meditating for the first group is 1, and the average times meditated for the second group is 2. Group 3 average times meditating was 4.95 times. Doing a yoga-based meditation once can help change how an HP feels while doing the meditation. However, repeated experiences with doing meditation are needed to effect systemic changes. We would expect group three to be more likely to show burnout and interoceptive awareness effects than groups 1 and 2.

Two of the six MAIA subscales were significantly different based on meditation frequency. Table 15 shows means and ANOVA statistics with three categories of meditation frequency as the independent variable and self-regulation and attention regulation as independent variables, both of which were significantly higher in group 3 (the HPs who did meditations more than twice). Planned comparisons showed that differences in interoceptive awareness were only detected among HPs who meditated more than twice. Simply attending the introduction webinar, and even doing a meditation once on their own, was not sufficient to impact interoceptive awareness. The meditations in the intervention were designed in part with a goal of activating and training interoceptive awareness. HPs who did the meditations more often reported directing attention to interoception more often, specifically, being significantly more likely to direct attention to bodily sensations as means of self-regulation and as a way to regulate attention.

Doing the meditations more often was associated with higher self-regulation and attention regulation. Although a direct relationship between doing the meditations and burnout was not detected, meditation frequency was associated with higher interoceptive awareness and higher interoceptive awareness was linked to lower burnout.

## 7. Discussion

This article was written for different audiences including designers of meditation interventions, and hospice organizations looking for ways to address HP burnout, and research scientists who study interoception or burnout or meditation or yoga or meditation interventions for health.

### 7.1. Understanding Yoga-Based Meditation

It is important that scientists who study meditation and hospice organizations considering burnout interventions pay attention to distinguishing characteristics of whatever specific meditation approach they are working with. Meditation is a diverse umbrella term. In addition to yoga-based meditation, there are other meditation approaches associated with contemplative traditions and underlying philosophies (such as Buddhism and Qigong). There are secularized adaptations of these practices (such as mindfulness meditation and MBSR). Additionally, there are all manner of other meditative and meditation experiences, teachers, and apps.

To provide clarity about the nature of the meditations used in the intervention, this article detailed the components of the six meditations in the intervention. We included the week 1 calming meditation video in the Supplementary Materials and explained how and why doing these meditations regularly might be expected to support mind–body integration. Meditation is fundamentally experiential. Reading a short explanation of a meditation does not convey what it is like to practice that form of meditation. Doing a meditation once provides much deeper insight into the nature of a meditation intervention than reading about it, although meditating once does not begin to convey the experience of meditating regularly over a long period of time.

### 7.2. Accessibility and Feasibility of the Intervention and Delivery Approach

Doing yoga-based meditation requires time and attention; meditation interventions for busy HPs need to be palatable, easy to do, and worthwhile. The meditation program and intervention delivery approach presented here were designed to encourage HPs throughout the hospice organization to do the meditations, with goals of promoting interoceptive awareness and combating burnout.

Feasibility and acceptability findings were encouraging. Half of the participants who experienced at least one meditation wanted to continue to have access to the meditations after the 6-week program ended. This suggests that they found the meditations valuable and had a desire to use them in the future. Two thirds of HPs who experienced at least one meditation went on to do a meditation on their own, a finding that suggests that HPs valued having access to these meditations. We know from focus group interviews with HPs that they did not want company-sponsored personal burnout interventions to be something time consuming that HPs were expected to do on their own time [2]. For example, some HPs mentioned not wanting to have to use personal time to attend company picnics or to complete online training modules. To be responsive to not burdening personal time, the intervention meditations were optional, under 12 min and could be done at home or at work. That the majority of meditation use occurred using the app on the HP’s workplace tablet or smartphone shows the importance of providing that option.

Introducing the meditation intervention at each team’s all-staff meeting worked for HPs who attended that meeting but left out HPs who missed that meeting. The internal app was also hard to find, so much so that, although it was available on every workplace tablet and smartphone, HPs did not discover it unless they were told it existed and shown exactly where and how to find it. A more prominent and easier to find app could encourage use even among HPs who miss the introductory session.

Huge differences were found in interest in long term access to them meditations based on teams, ranging for a low of 0% interest to a high of 86% interest. The team-based approach to delivery can provide opportunities for social support and may have encouraged use, especially if the operations manager or some of the team members were enthusiastic. Conversely, an unenthusiastic operations manager or a vocal nay-sayer could have the opposite effect. More research is needed on intervention implementation approaches to understand team-related factors that impact meditation adoption, as well as whether team-based or organization-wide deployment is preferable.

### 7.3. Interoceptive Awareness and Burnout

The findings supported the proposition that interoceptive awareness helps mitigate burnout. Here, we speculate on some of the ways that interoceptive awareness might work to reduce burnout in HPs.

The findings show that burnout is more malleable than permanent and that the three burnout factors are interdependent. We measured burnout at the beginning and end of the 7-week study period. Interpersonal disengagement was the most variable, such that only 13% of HPs who reported high interpersonal disengagement in either timeframe did so in both. Work exhaustion was the most stable burnout factor, with 55% of HPs reporting high work exhaustion in either time period, doing so for both. Professional fulfillment was fairly stable, with 43% of HPs reporting high professional fulfillment in either time period doing so in both. This malleability is good news for hospice organizations and intervention developers. The changes observed cannot be attributed to the intervention, but they do show that burnout is far from a permanent, unchangeable state.

The interdependence of burnout factors is also good news for mitigating burnout. Addressing one factor could help with the others. None of the HPs who scored high on professional fulfillment reported experiencing interpersonal disengagement. None of the HPs who scored low on work exhaustion reported experiencing interpersonal disengagement. Additionally, HPs who were low on work exhaustion were more likely to be high on professional fulfillment. Reducing work exhaustion could help increase professional fulfillment and reduce interpersonal disengagement.

Theories about how doing yoga-based practices improves the ways the human system responds to stress posit that interoception facilitates mind–body integration. High level brain processes (cognition) are better connected to bodily sensations and feelings, helping to improve conscious decision making. Additionally, low-level brain networks (the autonomic nervous system), become conditioned to calmer and less reactive physiological responses.

Why might interoceptive awareness help combat work exhaustion? When a HP is more aware of how she is feeling, she might take self-care steps such as taking a break, getting enough sleep, or going for a walk. She might make different choices, perhaps saying no to an assignment or approaching work tasks differently. A third possibility is that doing the meditations reduces reactivity to workload, mitigating the feeling of being overwhelmed.

Why might interoceptive awareness help combat interpersonal disengagement? Doing the meditations involved focusing attention on breath and body, which helps quiet the mind and relax the body. Being more connected with oneself can reduce reactivity in challenging interpersonal situations. Furthermore, the mediation objects in the intervention meditations were selected to be helpful in coping with challenging situations and people. In the “peaceful feeling” meditation, the participant supercharges seeds with a peaceful feeling, then drops a seed onto a relationship. The “releasing” meditation helps HPs release what they are holding on to (such as tension or tightness, or something upsetting someone said) that they no longer need. In the “cleansing waves” meditation, gentle waves wash up onto shore and then go back out. All of these practices can be helpful with difficult interpersonal situations.

Why might interoceptive awareness help increase professional fulfillment? When the mind is less agitated, we can see more clearly. Regular practice of the meditations may lessen focus on day-to-day problems and facilitate a big picture perspective more of the time. The bigger picture perspective allows for more effective identification of solutions to challenges or problems.

### 7.4. Meditation, Interoceptive Awareness, and Burnout

Meditation frequency matters. Meditating more than twice was associated with increased interoceptive awareness, specifically with significantly higher post-survey levels of self-regulation and attention regulation. Although the data did not show significant relationship between burnout and meditation frequency, research with a larger sample and an intervention delivery strategy that engaged more frequent meditation may find a relationship.

Future research should examine in detail how and when HPs choose to do the yoga-based meditations to manage how they feel. Research on the long term effects of meditation consistently show that salutatory effects increase over time with repeated meditation practice [51], but HPs in the study appeared to use the meditations when needed rather than regularly. HPs know that, due to their profession, they are at high risk of burnout. Focus group interviews with HPs at the same organization as the current study found that when HPs start noticing that they are feeling burned out, they take self-care steps such as mental health days to recover [2]. HPs indicated that workload and administrative demands were the major source of burnout, and that dealing with death and dying would periodically start to feel overwhelming, either because of a particularly distressing event or just unrelenting buildup [2]. The meditations in the intervention are tools HPs can use when life events present a need, such as before or after a stressful patient visit. Meditation can be done regularly, such as in the mornings before starting work. Alternatively, meditation can be done during particularly stressful times.

### 7.5. Study Limitations

The decision to make the meditation available via audio on an app automatically installed on hospice tablets and smartphones clearly facilitated access but introduced the limitation of relying on post-survey self-report rather than being able to automatically track meditation usage.

This field research was conducted in the context of busy HPs at a large hospice organization. The research team consisted of scientists who were external to the organization, and we were careful to make minimal time demands on clinical directors, operations managers, and HP clinical staff. Time and budget limitations precluded conducting interviews with the operations managers and focus group interviews with HPs who participated in the intervention would have yielded valuable qualitative data.

### 7.6. Effects of COVID-19 on the Research Process and Outcomes

The global pandemic severely impacted our research. Statewide COVID-19 restrictions interrupted data collection in midstream when the spring teams were nearing their 6th week. Research was halted (and, therefore, no post-surveys could be collected), drastically reducing our eventual sample size.

Unusual stressful external events during the study period not directly related to hospice work could have increased HP burnout and diminished professional fulfillment levels. Pre-surveys were collected in August 2020, and post-surveys in October. That time period covered the end of summer, the start of K-12 schools in hybrid or online only modalities due to COVID-19, increasing COVID-19 cases across the state, and the upcoming November 3 presidential elections.

Working from home, with children, partners, pets, and other interrupting factors may make it harder to find 12 uninterrupted minutes to do a meditation than while working at the office. Before COVID-19, HPs delivered care in person in patient homes, and worked from the regional office when not traveling to make patient visits. COVID-19 forced HPs to instantly shift from working at the office to working from their homes. The current research cannot answer the question of whether working from home during quarantine reduced meditation frequency.

### 7.7. Directions for Future Research

Given the positive feasibility and acceptability results and significant outcomes of the current study, future research with this intervention is warranted under more normal conditions after the global pandemic has ended. Future research should collect more detailed data about when, how often, and why HPs choose to do the meditation.

The yoga-based meditation program was administered within the hospice organizational structure. Clinical team operations managers arranged for the program to be presented at a monthly all-staff meeting, and then send emails to their teams each week throughout the program, introducing the new meditation for the week. The meditations were accessible on workplace tablets and smartphones without the need for internet access. The 12-min meditations could be done during working hours or on an individual basis.

The particular meditations were designed with HPs in mind. The duration and format as well as the specific and general intended outcomes were chosen to be accessible to and useful for HPs. There are myriad apps and web sites offering access to hundreds of yoga and meditation practices. Instead, this intervention offered only 6. As a result, uncertainty is reduced, and no time needs to be spent searching for the right meditation solution. Future research could compare adoption and use of access to generic meditations with a targeted intervention like the one studied here.

The intervention was intended to help HPs with managing stress. It may be more fruitful for hospice organizations to incorporate yoga-based meditations as tools in a sustained collection of resources for HPs to combat burnout, rather than a 6-week intervention that ends. Perhaps the meditations should always be available, with period events and reminders to re-animate use. Studies of long-term meditators consistently find that long term daily or frequent meditation has deeper and more systemic effects than the kinds of novice exposure the current research and most MBI intervention research studies [24,37,51,52]. Most HPs will not become daily meditators, but many may find these meditations to be helpful tools to support navigating the challenges of hospice work. Developing organizational approaches to sustain and increase engagement could amplify the value of MBIs in combatting HP burnout.

## 8. Conclusions

This study validated the feasibility and acceptability of the yoga-based meditation intervention and delivery approach and confirmed predicted relationships between yoga-based meditation, interoceptive awareness, and burnout.

The yoga-based meditations were valued and used by HPs. Half of those exposed to at least one meditation expressed a desire to continue to have access to the meditations after the 6-week program ended.

Nearly one-fifth of HPs who were exposed to the intervention at the all-staff meeting made the effort to do a meditation one time on their own, for a total of two meditation sessions. Nearly one-third experienced program meditations 3 to 5 times. Nine percent did meditations between 6 and 10 times.

Making the meditations available in an app that was placed on the workplace tablets and smartphones HPs use throughout the day for EMR updates and workplace communication, accessible without a need for internet ended up being the primary way HPs did the meditations. Fifty-eight percent of HPs who did a meditation on their own did so using their workplace tablet and 38% did so using their workplace smartphone.

Due to covid-19 work from home restrictions, three-fourth of HPs did a meditation at home, 29% in a car between patient visits (not while driving), and 23% at the office.

Introducing the 6-week program to individual clinical teams and doing the week 1 meditation together at their all-staff meeting gave HPs direct experience with what yoga-based meditations are like. Another benefit of deploying within a team was the social factor of being able to talk with co-workers about the experiences. A drawback of deploying at all-staff meetings was that HPs who missed the all-staff launch missed the intervention.

Higher interoceptive awareness was significantly related to lower burnout, particularly lower work exhaustion. Meditation frequency did not have measurable effects on burnout. Meditation frequency was linked to significantly higher self-regulation and attention regulation. Higher scores on four of six interoceptive awareness subscales were significantly related to lower burnout in HPs.

These findings affirm a role for interoceptive awareness training address burnout and provide validation of the yoga-based meditation intervention. Strategies to extend the intervention beyond six weeks and to engage in more meditation usage would likely increase benefits for HPs.

## Figures and Tables

**Table 1:** Movements and Postures of the Yoga-Based Meditations.

Movements, Postures	Meditations					
	Calming	Peaceful Feeling	Nature	Stability	Releasing	Cleansing Waves
Upright seated in chair, eyes closed	√	√	√	√	√	√
Arm extension up in front	√	√	√	√	√	√
Arms up from front then out to side				√		
Arm extension up in front alternating arms	√				√	√
Raise arms up from sides a little ways, a bit higher with each breath		√				
Arm extension from chest down to side	√					
Arm extension out from chest	√		√			
Arm extension out from chest, palms facing outward					√	
Hands on chest, arm extension down by sides						
Hands on opposite shoulders, arm extension down to legs				√		
Seated forward bend		√				√
Move hands over thighs toward knees	√					
Lean forward and back, finding position that feels most stable				√		

**Table 2:** Breath and Inhale:Exhale Ratios of the Yoga-Based Meditations.

Breathing and Inhale:Exhale Ratios	Meditations					
	Calming	Peaceful Feeling	Nature	Stability	Releasing	Cleansing Waves
Free observed breath	√	√	√	√	√	√
Align movement with breath	√	√	√	√	√	√
Extend exhale	√			√		
Pause after exhale	√				√	
On exhale, release what you no longer need					√	
On inhale, visualize a wave washing onto the shore. On exhale the wave goes back out, cleansing the shore.						√
Open hands on inhale, close hands on exhale						√

**Table 3:** Meditation Objects/Mental Focus of the Yoga-Based Meditations.

Meditation Objects	Meditations					
	Calming	Peaceful Feeling	Nature	Stability	Releasing	Cleansing Waves
Notice bodily sensations	√	√	√	√	√	√
Notice level of mental activity	√	√	√	√	√	√
Call to mind a peaceful feeling		√				
Imagine peaceful feeling as a handful of seeds. Pick up seed and drop onto one of your relationships		√				
Think of favorite place in nature			√			
Bring feeling of favorite place inside			√			
Feel self in favorite place in nature			√			
Connect with feeling of stability				√		
Release tension, tightness, what you no longer need					√	
Visualize a sandy beach with gentle waves						√

**Table 4:** Meditations and Principles in the 6-Week Program.

Week	Meditation	Principle
1	Calming	Yoga-based meditations are tools you can use to help change how you feel.
2	Peaceful Feeling	Checking in with yourself is a skill.
3	Place in Nature	Moving only as far as is comfortable is a skill.
4	Stability	Breath is an indicator and an influencer of the state of your system.
5	Releasing	Keep your cool by responding rather than reacting to events.
6	Cleansing Waves	Regular practice of meditation supports self-care.

**Table 5:** HP Roles (from the pre-survey).

Role	Percent
Nurse	43%
Aide	22%
Social Worker	7%
Chaplain/Spiritual Care	7%
Manager	5%
Administration	8%
Other	9%
n	126

**Table 6:** Meditation Frequency.

Times Played	n	Among All HPs	Among HPs Exposed to the Intervention
0	25	22%	
1	31	27%	34%
2	22	19%	24%
3 to 5	29	25%	32%
6 to 10+	9	8%	9%
	116		91

**Table 7:** Overall Pre- and Post- Survey Burnout Scores.

	Pre-Survey				Post-Survey			
	Mean	s.d.	% High	n	Mean	s.d.	% High	n
Professional Fulfilment	2.95	0.67	39%	76	2.78	0.75	32%	76
Work Exhaustion	1.30	0.84	38%	76	1.34	0.90	44%	75
Interpersonal Disengagement	0.71	0.68	21%	75	0.56	0.61	13%	72
Burnout	1.00	0.71	24%	75	0.94	0.69	25%	72

**Table 8:** Crosstab Comparisons of Pre- and Post- Survey Burnout.

**Professional Fulfillment (43% Stable)**			
	**Post-Survey**		
**Pre-survey**	**No**	**Yes**	**Total**
No	51% (n = 39)	8% (n = 6)	59%
Yes	20% (n = 15)	21% (n = 16)	41%
total	71%	29%	n = 76
**Work Exhaustion (55% Stable)**			
	**Post-Survey**		
**Pre-survey**	**No**	**Yes**	**Total**
No	47% (n = 34)	15% (n = 11)	61%
Yes	9% (n = 6)	29% (n = 21)	39%
total	56%	44%	n = 75
**Interpersonal Disengagement (13% Stable)**			
	**Post-Survey**		
**Pre-survey**	**No**	**Yes**	**Total**
No	69% (n = 50)	8% (n = 6)	78%
Yes	18% (n = 13)	4% (n = 3)	22%
total	88%	13%	n = 72

**Table 9:** Overall Pre- and Post- Survey Interoceptive Awareness Scores.

	Pre-Survey			Post-Survey		
	Mean	s.d.	n	Mean	s.d.	n
Self-Regulation	3.74	1.03	74	3.97	1.10	68
Attention Regulation	3.99	1.03	67	3.79	1.09	71
Emotional Awareness	4.71	1.02	69	4.70	1.04	75
Body Trusting	4.48	1.16	75	4.57	1.03	72
Body Noticing	4.23	1.04	70	4.42	1.02	69
Body Listening	3.69	1.35	75	3.73	1.21	72
Overall MAIA	4.15	0.79	64	4.24	0.96	61

**Table 10:** Post-Interpersonal Disengagement *t*-Tests.

	Fulfilled	Mean	s.d.	Significance
Body Trusting	NO	4.30	1.14	*t* = 3.579 (70), *p* = 0.005
	YES	5.28	0.70	
Pre-Work Exhaustion	NO	48%	50%	*t* = 2.928 (74), *p* = 0.005
	YES	14%	35%	
Pre-Interpersonal Disengagement	NO	30%	46%	(*t*-tests could not be conducted)
	YES	0%	0%	

**Table 11:** Post-Burnout *t*-Tests.

Scale	Burned out	Mean	s.d.	Significance
Self-Regulation	NO	4.22	1.03	*t* = 3.541 (65), *p* = 0.001
	YES	3.19	0.96	
Body Trusting	NO	4.85	0.98	*t* = 3.984 (68), *p* =0.000
	YES	3.72	1.16	
Body Listening	NO	3.94	1.20	*t* = 2.230 (68), *p* = 0.023
	YES	3.19	1.13	

**Table 12:** Post-Work Exhaustion *t*-Tests.

Scale	Exhausted	Mean	s.d.	Significance
Self-Regulation	NO	4.28	1.05	*t* = 2.823 (66), *p* = 0.006
	YES	3.56	1.03	
Attention Regulation	NO	4.24	1.19	*t* = 2.035 (65), *p* = 0.046
	YES	3.63	1.22	
Body Listening	NO	4.03	1.15	*t* = 2.376 (70), *p* = 0.020
	YES	3.36	1.20	
Pre-Professional Fulfillment (% high)	NO	44%	50%	*t* = 2.763 (73), *p* = 0.006
	YES	24%	44%	
Pre-Interpersonal Disengagement (% high)	NO	44%	50%	*t* = 4.51 (72), *p* = 0.001
	YES	5%	22%	

**Table 13:** Post-Interpersonal Disengagement *t*-Tests.

PFI Index	Disengaged	Mean	s.d.	Significance
Post-Professional Fulfillment (% high)	NO	35%	48%	(*t*-tests could not be conducted)
	YES	0%	0%	
Post-Work Exhaustion (% high)	NO	78%	44%	*t* = 2.410 (70), *p* = 0.019
	YES	37%	49%	

**Table 14:** Overall Meditation Frequency by Group.

Times Meditated	n	Mean	s.d.
once	27	1	0
twice	17	2	0
more	35	4.95	1.72

**Table 15:** Self-Regulation and Attention Regulation by Meditation Frequency.

Times Meditated	n	Self-Regulation		Attention Regulation	
		Mean	s.d.	Mean	s.d.
once	27	3.59	1.25	3.57	1.26
twice	19	3.78	0.98	3.69	1.05
more	35	4.24	0.88	4.27	1.12
		F = 4.519 (2, 78), *p* = 0.018		F = 4.218 (2, 76), *p* = 0.048	

## Data Availability

The dataset is available on Figshare, doi:10.6084/m9.figshare.14130590 (accessed on 27 November 2020).

## Supplementary Materials

The following are available online at https://www.mdpi.com/1660-4601/18/5/2515/s1, Video S1: Yoga Mind Tools calming meditation, s2, Video S2: Yoga Mind Tools Week 2 Principle: Checking in With Yourself is an Important Skill.

## References

[1] Cavanaugh K.J., Lee H.Y., Daum D., Chang S., Izzo J.G., Kowalski A., Holladay C.L. (2020). An Examination of Burnout Predictors: Understanding the Influence of job attitudes and environment. Healthcare.

[2] Lehto R.H., Heeter C., Forman J., Shanafelt T., Kamal A., Miller P., Paletta M. (2020). Hospice Employees’ Perceptions of Their Work Environment: A focus group perspective. Int. J. Environ. Res. Public Health.

[3] Dearmond I.M. (2013). The Psychological Experience of Hospice Workers during Encounters with Death. Omega.

[4] Lenzo V., Bordino V., Bonanno G.A., Quattropani M.C. (2020). Understanding the Role of Regulatory Flexibility and Context Sensitivity in Preventing Burnout in a Palliative Home Care Team. PLoS ONE.

[5] Parola V., Coelho A., Cardoso D., Sandgren A., Apóstolo J. (2017). Prevalence of Burnout in Health Professionals Working in Palliative Care: A systematic review. JBI Database Syst. Rev. Implement. Rep..

[6] Kamal A., Bull J.H., Wolf S.P., Swetz K.M., Shanafelt T.D., Ast K., Kavalieratos D., Sinclair C.T. (2020). Letter to the Editor Regarding “Prevalence and Predictors of Burnout Among Hospice and Palliative Care Professionals”. J. Pain Symptom Manag..

[7] Kamal A.H., Bull J.H., Wolf S.P., Swetz K.M., Shanafelt T.D., Ast K., Kavalieratos D., Sinclair C.T. (2020). Prevalence and Predictors of Burnout Among Hospice and Palliative Care Clinicians in the U.S. J. Pain Symptom Manag..

[8] Shanafelt T.D., Noseworthy J.H. (2017). Executive Leadership and Physician Well-being: Nine organizational strategies to promote engagement and reduce burnout. Mayo Clin. Proc..

[9] Kabat-Zinn J. (2009). Full Catastrophe Living: Using the Wisdom of Your Body and Mind to Face Stress, Pain, and Illness.

[10] Wahbeh H., Elsas S.-M., Oken B.S. (2008). Mind–body Interventions. Neurology.

[11] Burton A., Burgess C., Dean S., Koutsopoulou G.Z., Hugh-Jones S. (2017). How Effective are Mindfulness-Based Interventions for Reducing Stress Among Healthcare Professionals? A systematic review and meta-analysis. Stress Health.

[12] Bernstein A.M., Bar J., Ehrman J.P., Golubic M., Roizen M.F. (2014). Yoga in the Management of Overweight and Obesity. Am. J. Lifestyle Med..

[13] Shapiro S.L., Astin J.A., Bishop S.R., Cordova M. (2005). Mindfulness-Based Stress Reduction for Health Care Professionals: Results from a randomized trial. Int. J. Stress Manag..

[14] Gard T., Noggle J.J., Park C.L., Vago D.R., Wilson A. (2014). Potential Self-regulatory Mechanisms of Yoga for Psychologica lHealth. Front. Hum. Neurosci..

[15] Manotas M., Segura C., Eraso M., Oggins J., McGovern K. (2014). Association of Brief Mindfulness Training with Reductions in Perceived Stress and Distress in Colombian Health Care Professionals. Int. J. Stress Manag..

[16] The Best Meditation Apps (2020). The New York Times.

[17] Best Meditation Apps of 2020. https://www.healthline.com/health/mental-health/top-meditation-iphone-android-apps.

[18] Janes D. The Best Meditation Apps to Help with Anxiety. https://www.oprahmag.com/life/health/g29861798/best-meditation-apps/.

[19] Sakuma Y., Sasaki-Otomaru A., Ishida S., Kanoya Y., Arakawa C., Mochizuki Y., Seiishi Y., Sato C. (2012). Effect of a Home-based Simple Yoga Program in Child-care Workers: A randomized controlled trial. J. Altern. Complement. Med..

[20] Yang E., Schamber E., Meyer R.M.L., Gold J.I. (2018). Happier Healers: Randomized Controlled Trial of Mobile Mindfulness for Stress Management. J. Altern. Complement. Med..

[21] How to Meditate. https://www.headspace.com/meditation/how-to-meditate.

[22] Heeter C., Lehto R.H., Allbritton M., Day T., Wiseman M. (2017). Effects of a Technology-Assisted Meditation Program on Healthcare Providers’ Interoceptive Awareness, Compassion Fa-tigue, and Burnout. J. Hosp. Palliat. Nurs..

[23] Stamm B.H. (2014). The Concise ProQOL Manual. http://www.proqol.org/uploads/ProQOL_Concise_2ndEd_12-2010.pdf.

[24] Kirk U. (2011). Interoception Drives Increased Rational Decision-Making in Meditators Playing the Ultimatum Game. Front. Neurosci..

[25] Hill R.C., Dempster M., Donnelly M., McCorry N.K. (2016). Improving the Wellbeing of Staff Who Work in Palliative Care Settings: A systematic review of psychosocial interventions. Palliat Med..

[26] Dijxhoorn A.-F.Q., Brom L., van der Linden Y.M., Leget C., Raijmakers N.J. (2020). Prevalence of Burnout in Healthcare Professionals Providing Palliative Care and the Effect of Interventions to Reduce Symptoms: A systematic literature review. Palliat Med..

[27] Allbritton M., Heeter C. (2018). Meditation as an Intervention for Health: A framework for understanding meditation research. OBM Integr. Complement. Med..

[28] Desikachar T.K.V. (1982). The Yoga of T. Krishnamacharya.

[29] Desikachar T.K.V. (2001). The Viniyoga of Yoga, Applying Yoga for Healthy Living.

[30] Desikachar T.K.V. (2005). In Search of Mind.

[31] Heeter C., Allbritton M., Bossart C. (2019). Beyond Scientific Mechanisms: Subjective perceptions with viniyoga meditation. Int. J. Environ. Res. Public Health.

[32] Clarke T., Barnes P., Black L., Stussman B., Nahin R. (2018). Use of Yoga, Meditation, and Chiropractors among U.S. Adults Aged 18 and Over.

[33] Clarke T., Black L., Stussman B., Barnes P., Nahin R. (2015). Trends in the Use of Complementary Health Approaches among Adults: United States, 2002–2012.

[34] Farb N., Daubenmier J., Price C.J., Gard T., Kerr C., Dunn B.D., Klein A.C., Paulus M.P., Mehling W.E. (2015). Interoception, Contemplative Practice, and Health. Front. Psychol..

[35] Schmalzl L., Khalsa D.S. (2016). Research on the Psychophysiology of Yoga. The Principles and Practice of Yoga in Health Care.

[36] Desikachar T.K.V. (1999). The Heart of Yoga: Developing a Personal Practice.

[37] Deepak K.K. (2013). Yogic Intervention for Mental Disorders. Indian J. Psychiatry.

[38] Mohan A. (2002). Yoga for Body, Breath and Mind.

[39] Schmalzl L., Powers C., Henje Blom E. (2015). Neurophysiological and Neurocognitive Mechanisms Underlying the Effects of Yoga-Based Practices: Toward a comprehensive theoretical framework. Front. Hum. Neurosci..

[40] Schmalzl L., Powers C., Zanesco A.P., Yetz N., Groessl E.J., Saron C.D. (2018). The Effect of Movement-Focused and Breath-Focused Yoga Practice on Stress Parameters and Sustained Attention: A randomized controlled pilot study. Conscious. Cogn..

[41] Craig A.D. (2009). How Do You Feel—Now? The anterior insula and human awareness. Nat. Rev. Neurosci..

[42] Kerr C.E., Sacchet M.D., Lazar S.W., Moore C.I., Jones S.R. (2013). Mindfulness Starts with the Body: Somatosensory attention and top-down modulation of cortical alpha rhythms in mindfulness meditation. Front. Hum. Neurosci..

[43] Trockel M., Bohman B., Lesure E., Hamidi M.S., Welle D., Roberts L., Shanafelt T. (2018). A Brief Instrument to Assess Both Burnout and Professional Fulfillment in Physicians: Reliability and validity, including correlation with self-reported medical errors, in a sample of resident and practicing physicians. Acad. Psychiatry.

[44] Mehling W.E., Price C., Daubenmier J.J., Acree M., Bartmess E., Stewart A. (2012). The Multidimensional Assessment of Interoceptive Awareness (MAIA). PLoS ONE.

[45] Hölzel B.K., Carmody J., Vangel M., Congleton C., Yerramsetti S.M., Gard T., Lazar S.W. (2011). Mindfulness Practice Leads to Increases in Regional Brain Gray Matter Density. Psychiatry Res..

[46] Park C.L., Finkelstein-Fox L., Groessl E.J., Elwy A.R., Lee S.Y. (2020). Exploring How Different Types of Yoga Change Psychological Resources and Emotional Well-being across a Single Ses-sion. Complement. Ther. Med..

[47] Desikachar T.K.V. (2009). In Search of Mind.

[48] Bowen D.J., Kreuter M., Spring B., Cofta-Woerpel L., Linnan L., Weiner D., Bakken S., Kaplan C.P., Squiers L., Fabrizio C. (2009). How We Design Feasibility Studies. Am. J. Prev. Med..

[49] National Academy of Medicine (2020). Valid and Reliable Survey Instruments to Measure Burnout, Well-Being, and Other Work-Related Dimensions.

[50] Goleman D., Davidson R. (2017). The Science of Meditation: How to Change Your Brain, Mind and Body.

[51] Mocanu E., Mohr C., Pouyan N., Thuillard S., Dan-Glauser E.S. (2018). Reasons, Years and Frequency of Yoga Practice: Effect on emotion response reactivity. Front. Hum. Neurosci..

[52] Brefczynski-Lewis J.A., Lutz A., Schaefer H.S., Levinson D.B., Davidson R.J. (2007). Neural Correlates of Attentional Expertise in Long-term Meditation Practitioners. Proc. Natl. Acad. Sci. USA.

